# The Use of Flow Cytometry in Myelodysplastic Syndromes: A Review

**DOI:** 10.3389/fonc.2017.00270

**Published:** 2017-11-15

**Authors:** Laiz Cameirão Bento, Rodolfo Patussi Correia, Cristóvão Luis Pitangueiras Mangueira, Rodrigo De Souza Barroso, Fernanda Agostini Rocha, Nydia Strachman Bacal, Luciana Cavalheiro Marti

**Affiliations:** ^1^Hospital Israelita Albert Einstein—Clinical Pathology Laboratory, São Paulo, Brazil; ^2^Hospital Israelita Albert Einstein—Experimental Research Laboratory, São Paulo, Brazil

**Keywords:** myelodysplastic syndromes, flow cytometry, hematopoietic cell lineages, clonal disorder, diagnosis

## Abstract

Myelodysplastic syndromes (MDSs) are a heterogeneous group of hematopoietic stem cell diseases categorized by dysplasia in one or more hematopoietic cell lineages, as well as cytopenia and functional abnormalities in bone marrow cells. Several MDS classification methods have been proposed to categorize the disease and help professionals better plan in patients’ treatment. The World Health Organization classification, released in 2008 and revised in 2016, is the currently and the most used classification method worldwide. Recent advances in MDS molecular biology and innovations in flow cytometry have enabled the development of new parameters for MDS diagnosis and classification. Several groups have published flow cytometry scores and guidelines useful for the diagnosis and/or prognosis of MDS, which are mostly based on detecting immunophenotypic abnormalities in granulocyte, monocyte, and lymphoid lineages. Here, we review the current literature and discuss the main parameters that should be analyzed by flow cytometry with the aim of refining MDS diagnosis and prognosis. Furthermore, we discuss the critical role of flow cytometry and molecular biology in MDS diagnosis and prognosis, as well as the current challenges and future perspectives involving these techniques.

## Introduction

Myelodysplastic syndromes (MDSs) comprise a heterogeneous group of hematopoietic stem cell diseases with variable clinical courses that are characterized by dysplasia of one or more blood cell types, as well as cytopenia and functional abnormalities in bone marrow lineages (1–4). MDS is a progressive clonal disorder mostly affecting male adults between 60 and 70 years of age. It initiates as refractory anemia (RA) that may progress to a blastic phase (i.e., RA with excess blasts, RAEB) or even an acute myeloid leukemia (AML) (1–4). MDS initiates when damage to stem cells leads to complex alterations in daughter hematologic cells, including defects in cell differentiation, proliferation, and/or maturation (1–4).

Several MDS patient classification systems have been proposed to assist professionals in treatment planning. Between 1982 and 1985, MDSs were classified according to the French–American–British (FAB) organization, which is based on cell morphology and the number of blast cells in the bone marrow and/or peripheral blood (5). However, several cases did not fit within the FAB classification, and in 1997, the International Working Group for the Prognosis of MDS (IWG-PM) proposed the International Prognostic Scoring System (IPSS), which considers the number of blasts and cytopenia in the peripheral blood as well as the presence of cytogenetic abnormalities (6). In 2008, the World Health Organization (WHO) proposed their own classification (subsequently updated in 2016), which is currently used worldwide. The WHO 2016 updated version, modified the adult MDS terminology and terms such as “refractory anemia” and “refractory cytopenia” were replaced for MDS followed by the appropriate modifiers: single versus multilineage dysplasia, ring sideroblasts, excess number of blast cells, or the del(5q) cytogenetic abnormality. In addition, the classification includes revised diagnostic criteria for dysplasia and a more accurate method for the evaluation of blast numbers (7, 8). The WHO classification is considered an improvement over other MDS classifications (1, 3, 6–9).

Concurrent to progresses in the classification of MDS, advances in flow cytometry and molecular biology have contributed to the improved diagnosis, classification, and differentiation of MDS subgroups. Generally, MDS diagnosis is based on clinical history, peripheral blood and bone marrow cell morphology, cytogenetic data, and the exclusion of other diseases. However, some cases of peripheral cytopenia do not show obvious morphological abnormalities or bone marrow cytogenetic idiosyncrasies. For these specific cases, the differential diagnosis of clonal versus non-clonal disease with cytopenia has been a great challenge (2–4). An abnormal cytogenetic finding is an important indication of a clonal condition, but accounts for only 50% of the abnormalities observed in MDS patients (1, 7, 8). Although morphological analysis is considered indispensable for MDS diagnosis, flow cytometry has been an important tool for diagnosis, prognosis, and monitoring of the disease course. Furthermore, patients with RA, RA with ring sideroblasts, and patients with some cases of 5q syndrome may not display significant dysplastic morphology; however, immunophenotypic analysis can detect dysplastic characteristics in the myelomonocytic lineage. Therefore, an anomalous clone with an aberrant immunophenotype may display normal cell morphology (10). In our own work, we observed that MDS patients without altered monocyte morphology or cytogenetic abnormalities, but with a high percentage of monocytes with (abnormal) increased CD56 expression, exhibit functional alterations (unpublished data).

In March 2008, representatives from 18 European Institutes took part in a European LeukemiaNet workshop in Amsterdam, a first step toward establishing a standard approach for the use of flow cytometry in MDSs (11). Immunophenotyping by flow cytometry can identify abnormally increased and/or decreased population numbers, as well as aberrant expression of mature or immature lineage markers (12, 13). As a heterogeneous group of diseases, MDS does not have a specific antigen; thus, in 2008, the WHO suggested that diagnosis requires the presence of at least three immunophenotypic abnormalities (1, 2). This recommendation remains unchanged in the 2016 revised version (7–9).

To date, several groups have published flow cytometry scoring methods and guidelines for MDS diagnosis and/or prognosis, mostly based on the detection of immunophenotypic abnormalities during the maturation of granulocyte, monocyte, and lymphoid lineages (11, 14–17). For diagnosis, the classification system developed by Ogata et al. indicates MDS with scores ≥2 (15), and in terms of prognosis, clinicians frequently rely on the International Prognostic Scoring System (IPSS) and the Revised International Prognostic Scoring System (IPSS-R). The IPSS is based on the number of bone marrow blasts, cytogenetic abnormalities, and degree of cytopenia, which are used to define treatment and predict patients’ clinical response (9, 18). Another option is to use flow cytometry together with the IPSS-R. While there is currently no consensus regarding the prognostic power of flow cytometry, it has been shown that following allogeneic bone marrow transplantation, patients with increased phenotypic alterations have poorer prognosis and increased risk of relapse (18–21).

The aim of this review is to present all the main blood cell markers of MDS diagnosis and/or prognosis that should be the target of flow cytometry analyses. In addition, we discuss the critical role of flow cytometry and molecular biology in MDS diagnosis and prognosis, as well as the current challenges and future perspectives of this methodology.

## Evaluation of Dysplasia in Myeloid and B Lymphoid Progenitors

An important issue in MDS is blast quantification. Blast identification can be performed by flow cytometry using the classical combination of CD45^dim^ and SSC^low/int^ (11, 22). While blasts in MDS are usually restricted to this region (CD45^dim^ versus SSC^low/int^), maturation along the monocyte or neutrophil lineages may place abnormal blasts outside this region. Usually, number of blasts varies when using flow cytometry versus direct counts in morphological analysis, with flow cytometry yielding mostly higher counts. Even though diagnosis does not require the quantification of myeloid progenitor cells by flow cytometry, one should consider the cell source, since samples from hemodiluted bone marrow can compromise blast quantification (11, 13). CD45, CD34, CD117, HLA-DR, and CD123 are markers for myeloid progenitor cells (myeloblasts) and differentiate them from other cell populations, such as hematopoietic stem cells, B cell precursors, monoblasts, basophils, erythroblasts, and plasmacytoid dendritic cells (2, 11). MDS diagnosis and prognosis can also be elucidated *via* modified antigen expression patterns and the presence of abnormal proteins (23).

**Figure 1:**
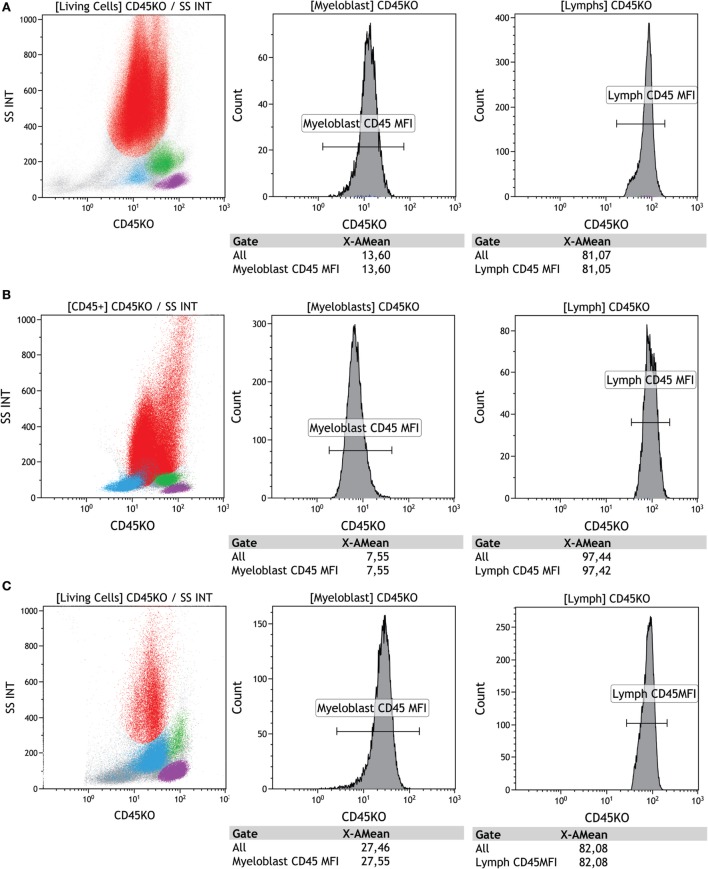
Evaluation of CD45 expression in bone marrow normal blasts versus dysplastic blasts. These populations first underwent gating of side scatter (SSC) versus CD45, followed by sequential analysis of myeloid progenitors (light blue) and lymphocytes (purple). Results are plotted in histograms. **(A)** SSC versus CD45 showing regular CD45 expression by myeloid progenitors (light blue) and peak of fluorescence (PF) of lymphocytes (purple) channel = 81.05 versus myeloblast (light blue) channel = 13.6 with regular ratio (81.05/13.6 = 5.95), **(B)** SSC versus CD45 showing weak CD45 expression by myeloid progenitors (light blue) and PF of lymphocytes (purple) channel = 97.42 versus myeloblast (light blue) channel = 7.55 with altered ratio (97.42/7.55 = 12.9), **(C)** SSC versus CD45 showing strong CD45 expression by myeloid progenitors (light blue) and PF of lymphocytes (purple) channel = 82.08 versus myeloblast (light blue) channel = 27.55 with altered ratio (82.08/27.55 = 2.98). SSC versus CD45 highlight the populations of granulocytes (red), monocytes (green), lymphocytes (purple), and progenitors and precursors (light blue). Samples were acquired with a Navios flow cytometer and analyzed using Kaluza software (Beckman Coulter).

Dysplasia in immature myeloid lineages can be verified by the absence or decreased expression of CD45 and CD117 (2, 16). CD45 expression in normal bone marrow blasts versus dysplastic blasts can be evaluated first by measuring the side scatter (SSC) versus CD45 gate, followed by sequential analysis of myeloid progenitors and lymphocytes plotted in a new histogram graph (Figure 1). CD45 expression in progenitor cells is obtained by dividing the peak of fluorescence (PF) of CD45 in the lymphocyte population by the PF of CD45 in the myeloblast population as follows:

$$\text{Equation}=\frac{\text{PF of CD45 expression in lymphocytes}}{\begin{array}{c} {\text{PF of CD45 expression in CD34}}^{\text{+}}\\ \text{cells in myeloblast related clusters} \end{array}}\text{.}$$

**Figure 2:**
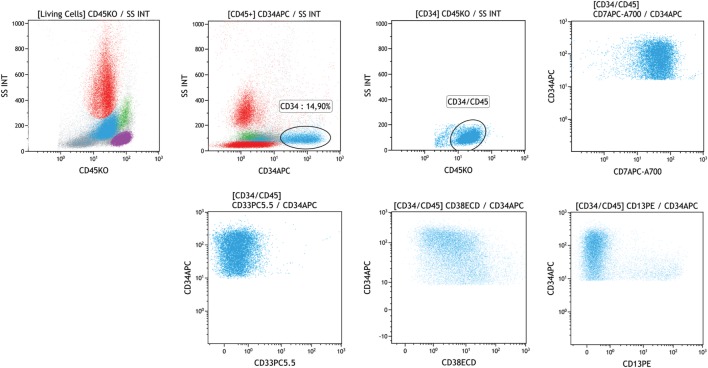
Dysplasia evaluation in myeloid progenitor cells. Leukocytes were identified by SSC versus CD45. Enhanced percentage of CD34 (14.9%) (light blue) was identified through sequential gates with abnormal expression of CD7, absence of CD33 and CD13, and weak expression of CD38. SSC versus CD45 highlights the populations of granulocytes (red), monocytes (green), lymphocytes (purple), and progenitors and precursors (light blue). Samples were acquired with a Navios flow cytometer and analyzed using Kaluza software (Beckman Coulter).

As shown in Figure 1, values <4 or >7.8 add 1 point to the Ogata Score (15). For myeloblasts, CD45 values <4 reflect low expression and values >7.8 indicate high expression. However, these values are not as well defined for pediatric or hypocellular samples and can, therefore, not be used for diagnosis (24). Other possible indicators of MDS are increased HLA-DR/CD34 ratios, decreased or absent CD38 expression on CD34 progenitor cells, and increased myeloblast frequency (CD34^+^/CD19^−^) (Figure 2); also, increased myeloblast percentage values (≥2%) add 1 point to the Ogata Score (15).

The increased or modified pattern of CD34 expression and the absence of CD33 or CD13 are also considered criteria for dysplasia (Figure 2). This analysis should also be performed in the CD117^+^/CD34^+^ population, because some dysplastic precursors may also fail to express CD34 (2). Moreover, patients who display myeloid progenitor cells with enhanced CD117 expression are known to have poor prognosis (18).

Dysplasia is characterized by maturative asynchronism in the expression of CD10, CD11b, and CD15 on CD34 positive cells, as well as abnormal expression of CD2, CD5, CD7, and CD56 in the CD34 population (see Figure 2) (2, 11, 25). CD7 expression, however, should be evaluated carefully, since there is a small population of regular precursors that may express this marker, especially in recovering bone marrow (22, 25). Furthermore, anomalous lymphoid marker expression in myeloid blasts is associated with poor prognosis; patients with this expression display resistance to Azacytidin treatment and are more dependent on blood transfusions (18). A reduced expression of CD10 and CD15 in neutrophils is more prevalent in low-risk MDS patients, and CD15 expression is associated with better prognosis than CD7 expression (19).

**Figure 3:**
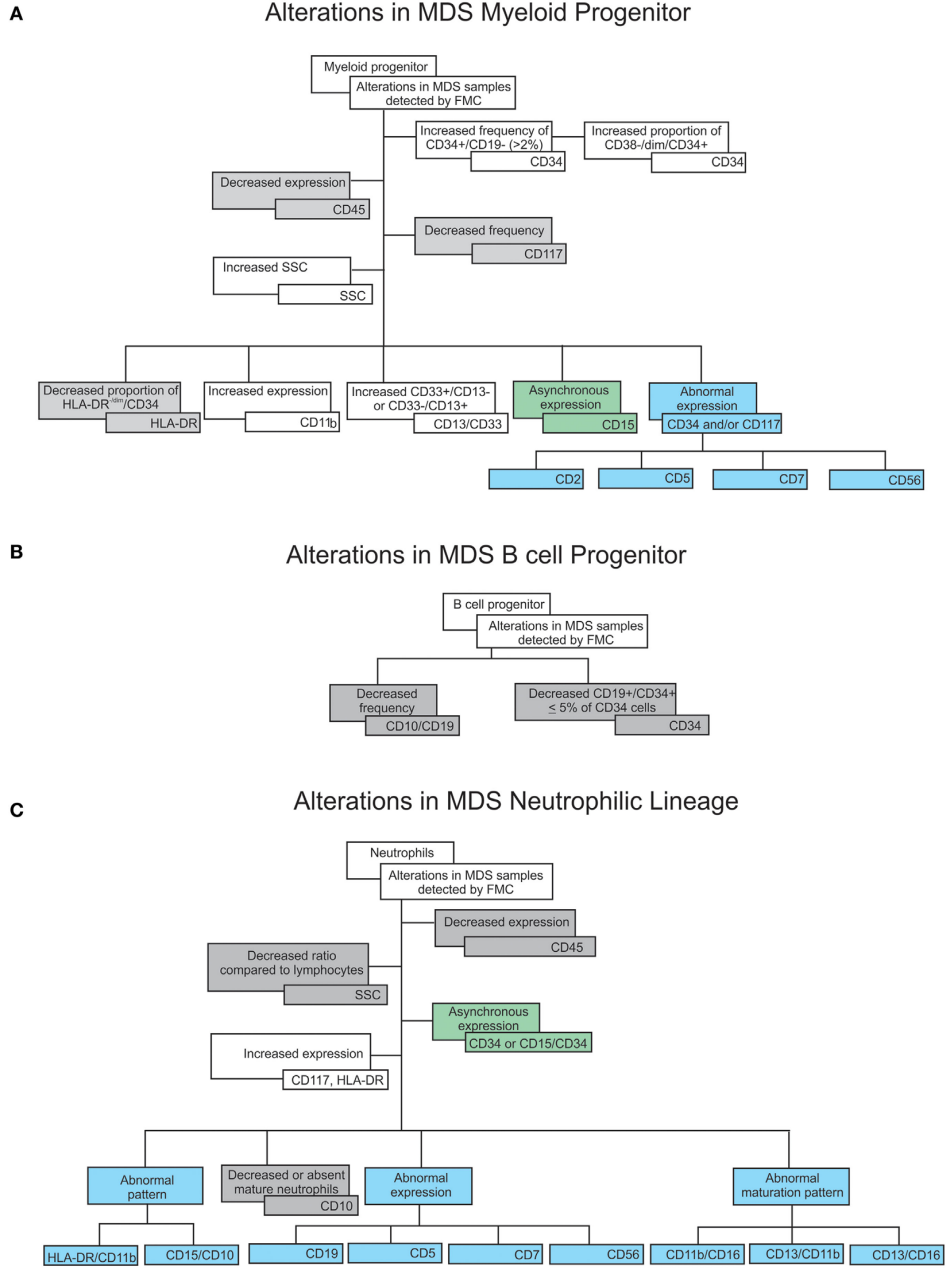

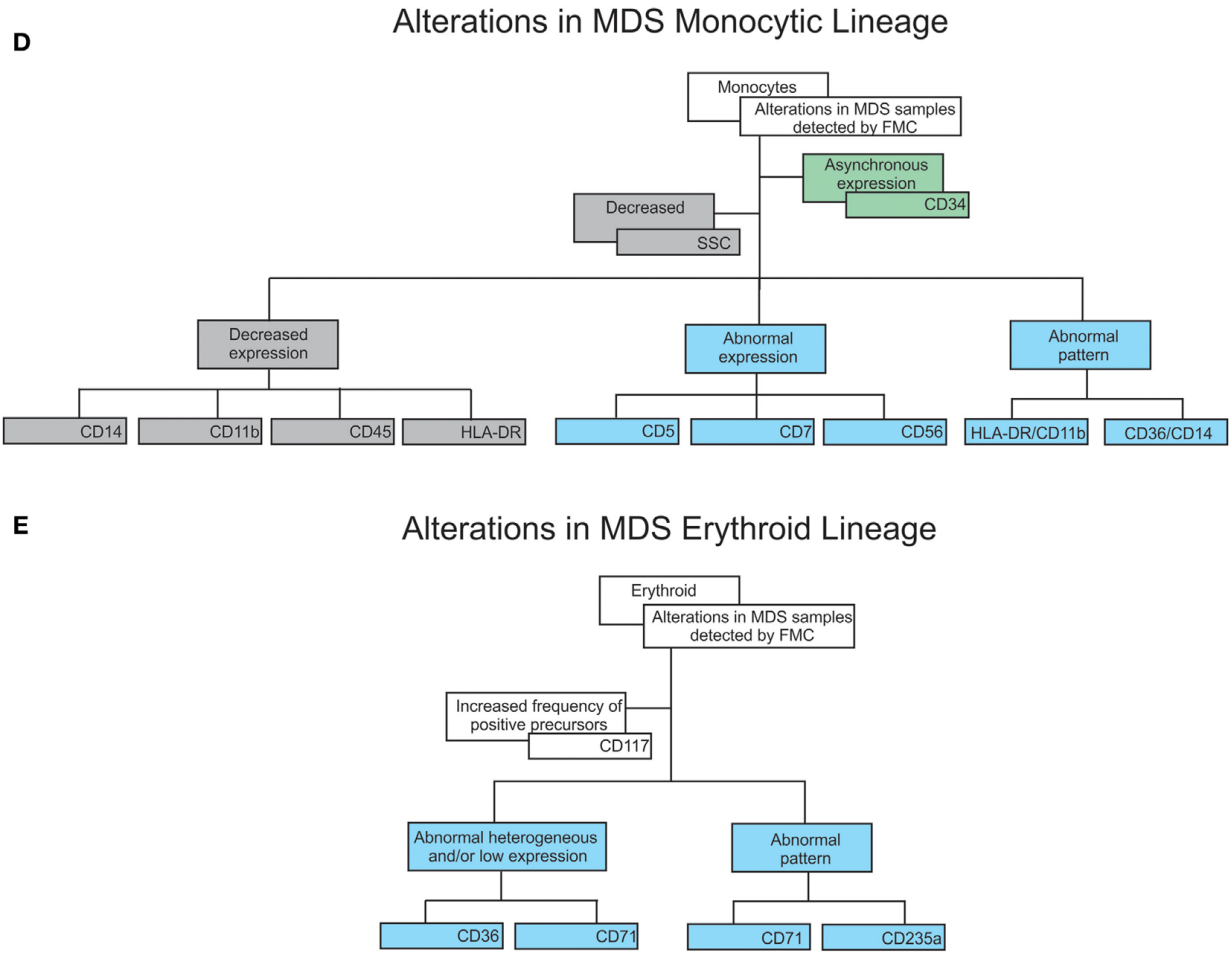
Flowcharts summarizing cell dysplastic characteristics verified by flow cytometry in myelodysplastic syndromes (MDS) patient samples: increased expression, frequency, or proportion are displayed in the white boxes; decreased expression, frequency, or proportion are displayed in the grey boxes; asynchronous expression (expression of immature markers together with lineage markers in the same cell) displayed in the green boxes; aberrant expression (as an example, myeloid cells expressing other lineage markers) displayed in the blue boxes, all compared to healthy samples. These alterations are separated by lineage as follows: **(A)** alterations in myeloid progenitors, **(B)** alterations in B progenitor cells, **(C)** alterations in the neutrophilic lineage, **(D)** alterations in the monocytic lineage, and **(E)** alterations in the erythroid lineage.

Yet another important marker of MDS is B lymphoid progenitor cells, which are rare in MDS; values ≤5% add 1 point on the Ogata Score. These cells can be identified by the CD45^dim^ and SSC^low/int^ gate together with CD34, CD19, and CD10 markers (11, 14, 26, 27). Even though low numbers of B cell progenitors are indicative of dysplasia, other myeloproliferative diseases can also exhibit low numbers of this cell population (11). A summary of the main lineage alterations and minimal requirements recommended for assessing dysplasia of myeloid and lymphoid progenitors is shown in Figures 3A,B.

## Evaluation of Dysplasia in Neutrophils

**Figure 4:**
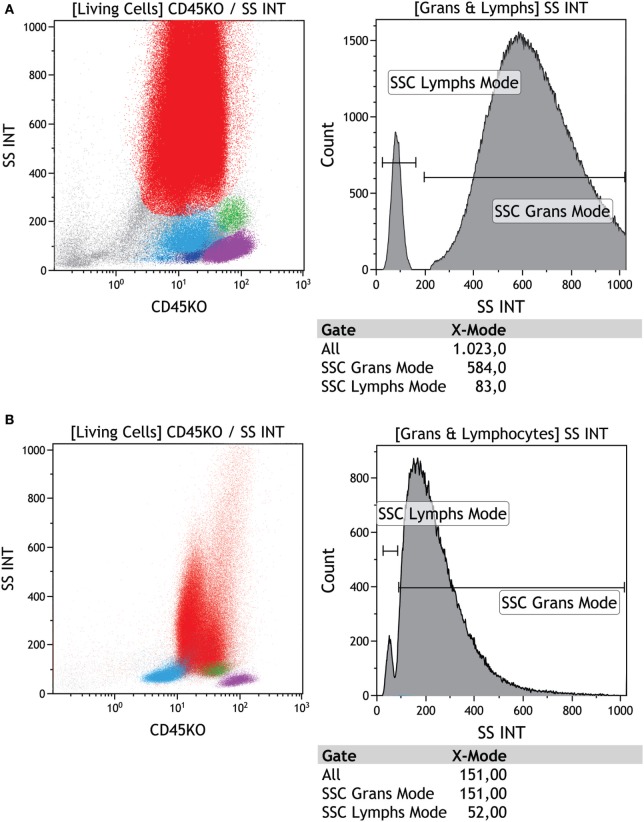
Evaluation of granularity in a regular versus dysplastic sample using side scatter (SSC). **(A)** First dot plot shows the leukocyte population visualized by SSC versus CD45 and the second plot shows the SSC peak of fluorescence (PF) in neutrophils (red) = 584.00 versus lymphocytes (purple) = 83.33 (584.00/83.33 = 7.03); this is a normal ratio, **(B)** first dot plot shows SSC versus CD45 and the second plot shows the SSC PF in neutrophils (red) = 151.00 versus lymphocytes (purple) = 52.00 (151.00/52.00 = 2.90); this is an altered (patient) ratio. SSC versus CD45 highlights the populations of granulocytes (red), monocytes (green), lymphocytes (purple), and progenitors and precursors (light blue). Samples were acquired with a Navios flow cytometer and analyzed using Kaluza software (Beckman Coulter).

Neutrophils are commonly affected in MDS and flow cytometry is often used to analyze the CD45^int^ versus SSC combination and to identify mature neutrophils (2, 28). CD33 differential expression can be used to distinguish between neutrophils (weak expression) and monocytes (strong expression) (11). In MDS, the most frequent dysplastic characteristic in granulocytes is reduced granularity in neutrophils, which can be verified by flow cytometry and is associated with poor prognosis (18, 29). This alteration can be assessed by dividing the granulocyte scatter peak channel by the lymphocyte scatter peak channel (Gra/Ly SSC ratio) (Figure 4). This strategy minimizes intra-operator variability, and Gra/Ly values ≤6.0 reflect granulocytes with reduced internal complexity, which adds 1 point to the Ogata Score (14, 15).

**Figure 5:**
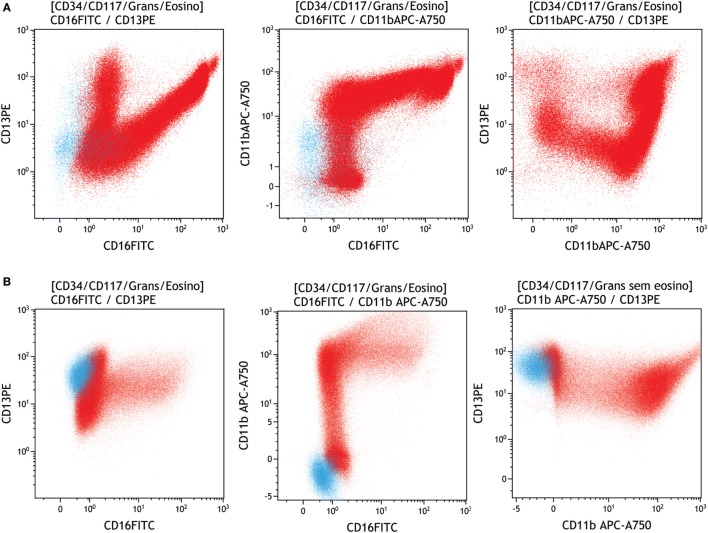
Evaluation of neutrophil maturation in a regular versus dysplastic sample. Neutrophils were identified and gated (red) on SSC versus CD45 and myeloid progenitors (light blue), and were displayed in the following plots: **(A)** regular pattern in a neutrophil (red) maturation curve according to the expression of CD13/CD16, CD11b/CD16, and CD13/CD11b, **(B)** abnormal pattern in a neutrophil (red) maturation curve according to the expression of CD13/CD16, CD11b/CD16, and CD13/CD11b. Samples were acquired with a Navios flow cytometer and analyzed using Kaluza software (Beckman Coulter).

Dysplastic granulocytes can also be identified by enhanced or diminished expression of CD45, CD11b, CD13, CD16, CD33, and CD64. Another very frequent characteristic of granulocyte dysplasia are alterations in the following ratios: CD13/CD11b, CD13/CD16, and CD11b/CD16 (more frequently, CD16), which appear as abnormal patterns on the maturation curve. These abnormal maturation patterns reflect a reduction in the number of mature granulocytes (see Figure 5 for an example) (2, 13, 23, 30).

**Figure 6:**
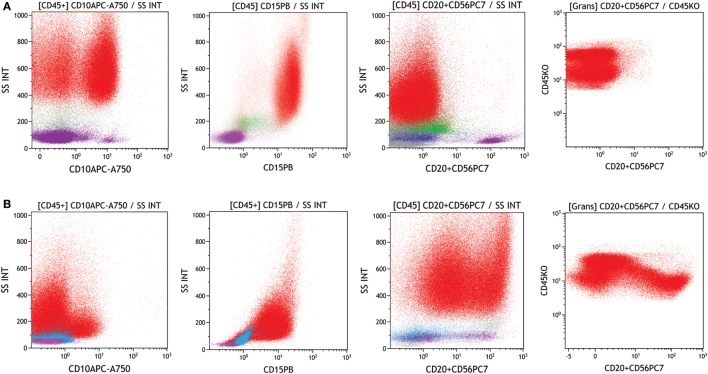
Evaluation of neutrophils with abnormal expression in a regular versus dysplastic sample. Neutrophils were identified and gated (red) on SSC versus CD45. **(A)** Regular bone marrow showing the CD10 and CD15 expression in neutrophils (red) and no abnormal CD56 expression, **(B)** myelodysplastic syndromes bone marrow showing diminished CD10 and CD15 expression in neutrophils (red) and abnormal expression of CD56. Samples were acquired with a Navios flow cytometer and analyzed by Kaluza software (Beckman Coulter).

Mature granulocytes with diminished or absent CD10 expression, abnormal CD10 pattern and anomalous CD10/CD15 ratios indicate dysplasia (27). Moreover, granulocytes with abnormal expression of lineage markers such as CD5, CD7, CD19, and CD56, should also be analyzed (see Figure 6) (16).

In some genetic polymorphisms, the expression of markers such as CD33 may be diminished but not abnormal (2). Also, since CD16 is associated with glycosylphosphatidylinositol, a lack of CD16 may also be observed in paroxysmal nocturnal hemoglobinuria (31, 32). A summary of the main alterations observed in neutrophils as well as the requirements for assessing neutrophil dysplasia are shown in Figure 3C.

## Evaluation of Dysplasia in Monocytes

Analyzing dysplasia in monocytes by conventional morphology is a challenge. Nowadays, monocyte characterization by flow cytometry is based on CD45 expression (moderate-strong) and *SSC* (moderate) together with markers such as CD14, CD33, CD64, and HLA-DR (23, 33). Monocyte quantification based only on CD14 expression is not recommended due to possible underestimation, especially when monocyte progenitors are present (2).

**Figure 7:**
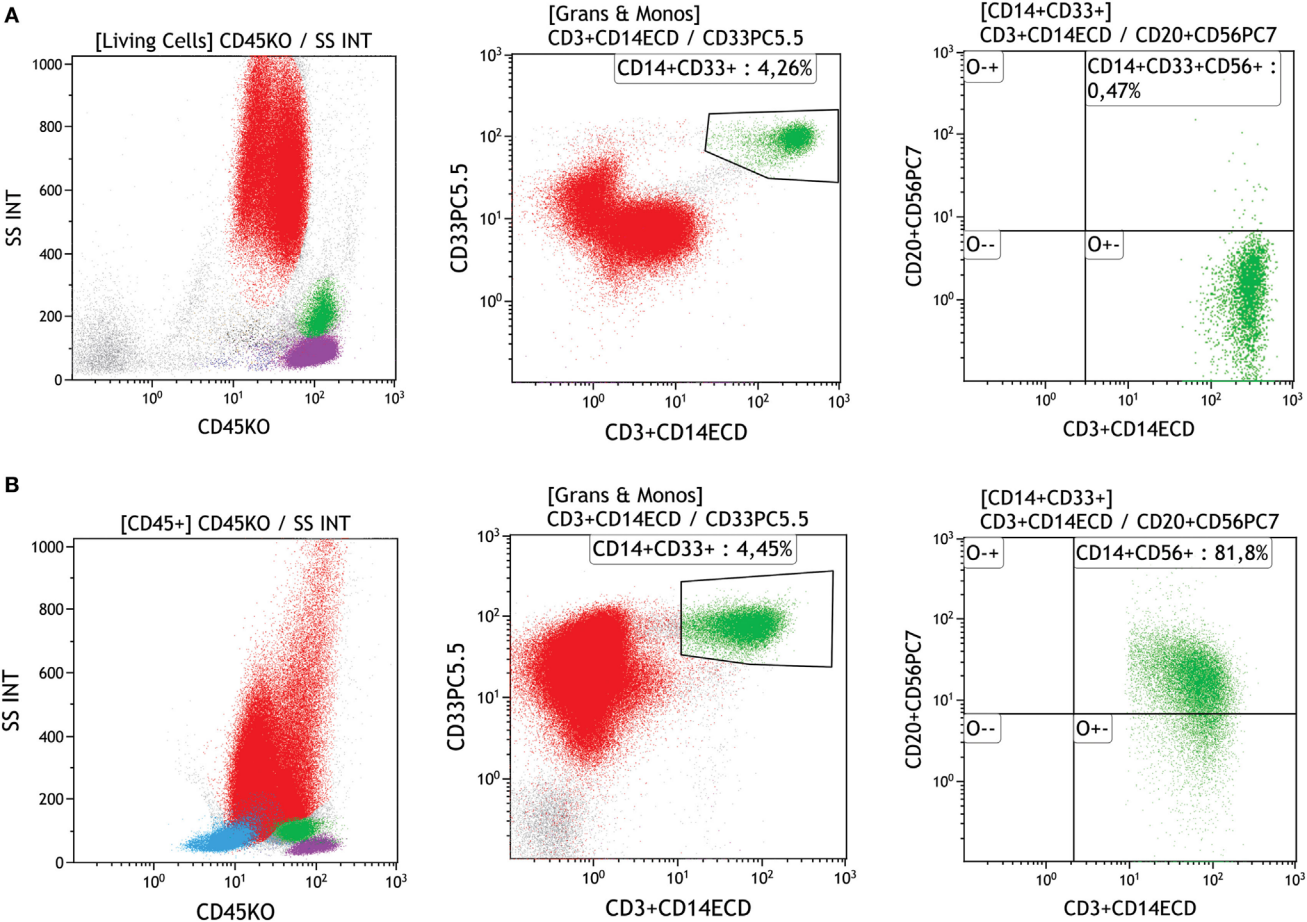
Evaluation of monocytes with abnormal expression in a regular versus a dysplastic sample. **(A)** First dot plot shows a leukocyte population on side scatter (SSC) versus CD45; the monocyte gate was performed on a second plot that displays CD33 versus CD14 showing mature monocytes (green) and the third plot displays CD56 versus CD14 and shows absence of CD56 expression on monocytes (green) in a regular sample, **(B)** first dot plot shows SSC versus CD45, the second plot shows the CD33 versus CD14 showing mature monocytes (green), and the third plot displays CD56 versus CD14 and shows the abnormal CD56 expression in a monocyte (green) population. Samples were acquired with a Navios flow cytometer and analyzed using Kaluza software (Beckman Coulter).

Monocytes display immunophenotypic alterations in MDS, and depending on gate strategy, the presence of granulocytes with low SSC can interfere in monocyte analysis (18, 30). The most frequent monocyte alterations in MDS are (1) increased or decreased monocyte number, (2) abnormal intensity of CD13, CD33, CD14, CD36, CD45, or CD64 expression, and (3) altered CD11b/HLA-DR or CD36/CD14 ratios (33). CD13 is involved in inflammatory responses during cell differentiation, proliferation, and adhesion, and monocytes with low CD13 expression are associated with good prognosis in MDS. Pro-inflammatory monocytes display enhanced CD13 expression, which may contribute to the regulation of other immune cell subsets. Taken together, monocytes with abnormal CD13 expression play a positive role in MDS (18). MDS patients may also display maturative asynchronism with the simultaneous presence of CD34 and CD14 and abnormal expression of CD56, CD2, CD7, and CD19 (2, 11). Yet abnormal CD56 expression may also occur in chronic myelomonocytic leukemia, in recovering bone marrow after chemotherapy or transplantation, and during infections. Since activated monocytes can express CD56, this expression is considered abnormal if ≥20% (see Figure 7) (2, 11, 23, 34–36). A summary of this lineage’s main alterations and minimal requirements for monocyte dysplasia is shown in Figure 3D.

## Evaluation of Dysplasia in the Erythroid Lineage

Dysplasia in the erythroid lineage can be defined by weak or negative CD45 expression and low FSC versus SSC (37). Currently, very few known antibodies are available to evaluate this lineage, and very few studies in MDS focus on the erythroid compartment (2, 11).

A study published in 2013 showed that approximately 88% of MDS patients have dysplastic alterations in the erythroid lineage (5, 37). Although erythroid lineage development and flow cytometry patterns were described by Loken et al. in 1987 (37), only in 2001 did investigators report abnormalities found in this lineage by flow cytometry (29).

The erythroid lineage markers usually analyzed by flow cytometry include CD36 (thrombospondin receptor), CD71 (transferrin receptor), CD235a (glycophorin-A), CD105, and CD117 (2, 16).

**Figure 8:**
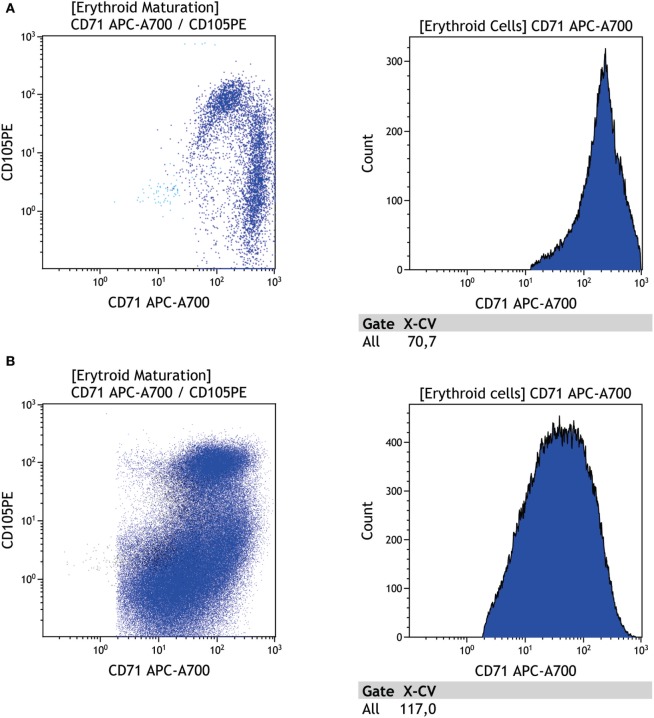

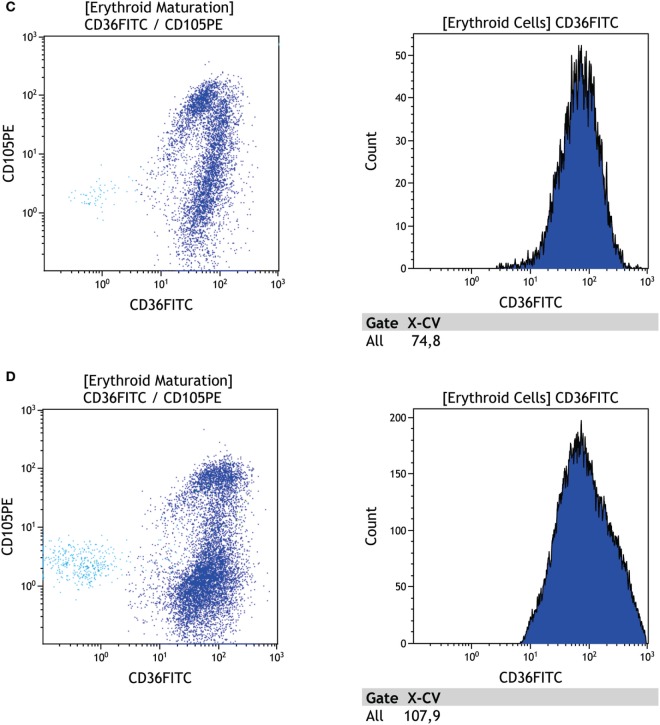
Evaluation of the erythroid lineage maturation curve in a regular versus dysplastic sample. **(A)** First dot plot shows erythroid population (blue) CD105 versus CD71, the second plot shows CD71 fluorescence intensity coefficient of variation = 70.7 on a regular sample, **(B)** first dot plot shows erythroid population (blue) CD105 versus CD71, the second plot shows CD71 fluorescence intensity coefficient of variation = 117.1 on a dysplastic sample, **(C)** first dot plot shows erythroid population (blue) CD105 versus CD36, the second plot shows CD36 fluorescence intensity coefficient of variation = 74.8 on a regular sample, **(D)** first dot plot shows erythroid population (blue) CD105 versus CD36, the second plot shows CD36 fluorescence intensity coefficient of variation = 107.9 on a dysplastic sample. Samples were acquired with a Navios flow cytometer and analyzed using Kaluza software (Beckman Coulter).

Erythroid lineage dysplasia in MDS is frequently manifested as an immature population of CD117 erythrocytes and/or increased or reduced CD105. However, these features are not MDS specific and should be evaluated with caution since cellular lysis may affect the number of erythroid progenitors (23, 38). Maturative asynchronism in CD71 versus CD235a and low CD36 and CD71 expression have been reported by a few authors; since platelets express CD36, this characteristic should be evaluated with care (21, 29, 38, 39). Moreover, the enhanced number of nucleated erythroid cells relative to the total number of nucleated cells and enhanced CD105 expression could be considered a characteristic of dyserythropoiesis. When comparing the expression of four erythroblast surface markers between 53 undeniable MDS patients and 46 control participants, Mathis et al. identified that the coefficients of variation (CV) of the fluorescence intensity of CD71 and CD36 are highly sensitive and robust discriminative tools for MDS diagnosis (see Figure 8) (38). These two parameters, together with low hemoglobin levels, make up the RED score (values ≥3 suggest MDS) (38).

Recently, the European Leukemia Net published the results of a multicenter study on immunophenotypic characterization of the erythroid lineage in MDS patients. In that study, MDS patients showed an enhanced number of cells expressing CD71, while the mean fluorescence intensity of CD71 and CD36 was lower than that of a control sample. On the other hand, the CD36 and CD71 coefficient of variation (CV) was relatively enhanced, corroborating findings by Mathis et al. (38, 40). Altogether, these results provide tools to facilitate discrimination between patients with MDS and patients with non-clonal cytopenia. Also in line with Mathis et al., Cremers et al. showed that the immunophenotypic analysis of the erythroid lineage in MDS patients has high sensibility with no or low compromise of specificity (38, 41). A summary of the main alterations in this lineage and the minimal requirements for assessing erythroid dysplasia are shown in Figure 3E.

## The Prognostic Value of Flow Cytometry

As mentioned above, an anomalous phenotype found by flow cytometry may be associated with poor prognosis in MDS patients. Furthermore, IPSS and IPSS-R are often used to establish treatments and to estimate clinical outcomes (9, 42). The flow cytometry score system (FCSS) measures immunophenotypic abnormalities in myelomonocytic cells and myeloid cells. The higher the number of myeloid progenitors with abnormalities, the higher the FCSS score. High FCSS scores are associated with an adverse diagnosis as well as higher chances of relapse after allogeneic transplantation (22). Studies have shown that patients with various phenotypic abnormalities have relatively poorer prognosis even when classified as low-risk MDS by IPSS and IPSS-R (43). A recent study by Alhan et al. showed that patients with decreased SSC have relatively worse prognoses (43). Another abnormality associated with a poorer prognosis is the increased expression of CD117 in myeloid progenitors, which enables malignant clone survival. On the other hand, patients with decreased CD13 expression in mature monocytes show better prognosis. No studies thus far have shown whether flow cytometry can aid in the prognosis of patients diagnosed with refractory MDS with single lineage, MDS refractory with ring sideroblasts and unclassifiable MDS (24).

## Diagnosis and Flow Cytometry: Future Perspectives

Relative to our knowledge regarding AML, the general understanding of the molecular pathology underlying MDS remains considerably primitive. Genetic sequencing has greatly contributed to our knowledge regarding the role of genetic mutations in MDS pathogenesis and progression, including abnormal RNA splicing and DNA methylation, altered transcription factors, and alterations in signal transduction (44). However, many of these genic alterations are heterogeneous and do not contribute to MDS classification or diagnosis. Mutations that could contribute to MDS classification are advancing gradually, and are correlated with morphological analysis, cytogenetics, and immunophenotypic alterations (45, 46). Additionally, a deeper investigation of immunophenotypic alterations may lead to focused genic alterations that could be used as a new target for drug development.

## Conclusion

Flow cytometry is an important tool in diagnosing MDS and can provide information not obtained through morphological, cytogenetic, or molecular biology analyses. However, flow cytometry still faces a few challenges, including a lack of consensus regarding the most appropriate parameters to be analyzed and lack of a specific marker that discriminates MDS from other pathologies. Another challenge is the study of erythroid series that do not have a specific lineage marker. Thus, future multicentric studies should aim to determine which parameters are more informative in discriminating MDS from other forms of cytopenia to improve both diagnoses and prognoses. Moreover, establishing standard techniques would contribute to greater comparability across studies.

## Author Contributions

LB wrote the article; RC, CM, RB, FR, and NB reviewed the article; LM wrote and reviewed the article.

## Conflict of Interest Statement

The authors declare that the research was conducted in the absence of any commercial or financial relationships that could be construed as a potential conflict of interest.
